# Bayesian Optimization for Efficient Multiobjective Formulation Development of Biologics

**DOI:** 10.1021/acs.molpharmaceut.5c00591

**Published:** 2025-09-26

**Authors:** Isabel Waibel, Timo N. Schneider, Fiona J. Fischer, Poonpat Dumnoenchanvanit, Alina Kulakova, Tin Duy Nguyen, Thomas Egebjerg, Søren Bertelsen, Nikolai Lorenzen, Paolo Arosio

**Affiliations:** † Department of Chemistry and Applied Biosciences, Institute for Chemical and Bioengineering, ETH Zürich, Vladimir-Prelog-Weg 1-5/10, Zürich 8093, Switzerland; ‡ Novo Nordisk A/S, Therapeutics Discovery, Novo Nordisk Park, Måløv 2760, Denmark; § Novo Nordisk A/S, Digital Science & Innovation, Novo Nordisk Park, Måløv 2760, Denmark

**Keywords:** Bayesian optimization, machine learning, multiobjective optimization, monoclonal antibodies, protein formulation, excipients, developability

## Abstract

Biologics, including emerging engineered formats, can often exhibit poor developability profiles, complicating their translation into successful therapeutics. While formulation design can substantially mitigate some developability issues, it represents a highly complex optimization challenge due to the need to simultaneously improve multiple biophysical properties, navigate a vast design space, and account for nonlinear or synergistic interactions among excipients. Traditional design of experiments methods can reduce experimental effort but are limited by difficulties in managing high-order complexities and a propensity to become trapped in local optima. In response, machine learning techniques combined with (high-throughput) screenings have emerged as powerful strategies to overcome these limitations, dramatically reducing the number of required experiments. The ability of these models to capture nonlinear relationships and interactions among multiple features enables efficient navigation in a high-dimensional design space. We present a combined Bayesian optimization and experimental screening method that concurrently optimizes three key biophysical properties of a monoclonal antibody—melting temperature *T*_m_, diffusion interaction parameter *k*_D_, and stability against air–water interfaces. We demonstrate its effectiveness through the identification of highly optimized formulation conditions in just 33 experiments. Furthermore, our approach can account for essential formulation constraints such as osmolality and pH, ensuring practical applicability. We show that beyond optimization, our method provides valuable insights into the influence of individual excipients on each biophysical property across formulations. Furthermore, it highlights the need to balance trade-offs between conflicting properties, such as the opposing effects of pH on *T*_m_ and *k*_D_.

## Graphical abstract

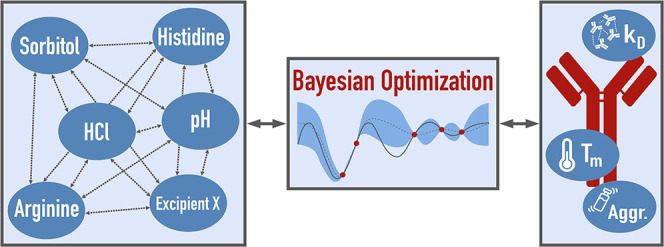

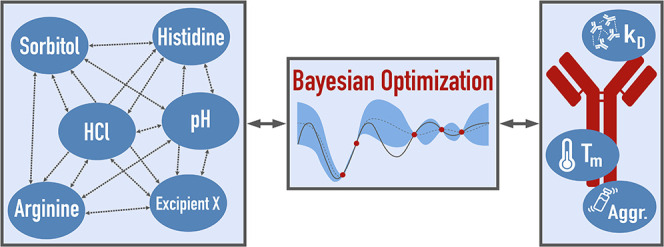

## 1 Introduction

Biologics are playing an increasingly central role in modern medicine, with monoclonal antibodies (mAbs) representing their largest class.^1^ To date, 115 mAbs have been approved by the FDA, addressing a broad spectrum of diseases such as cancer, autoimmune disorders, and infections.^2^ Additionally, engineered mAb formats such as bispecifics and fragments are emerging, with 20 constructs already approved by the FDA.^2^ These next-generation mAb formats offer several advantages over conventional mAbs, including enhanced diffusion rates, reduced manufacturing costs, and the potential for innovative therapeutic strategies.^3–5^

Although the initial focus in the development of biologics is their therapeutic function, a set of physicochemical properties globally referred to as developability are crucial to define the potential of a biologic candidate to evolve into a manufacturable, safe, and effective drug.^6–10^ Early and efficient evaluation of developability is key to avoiding costly delays and ensuring that biologic candidates move smoothly through the complex, resource-demanding development process. With respect to conventional mAbs, engineered antibody formats often experience higher developability issues, likely due to their lack of natural evolutionary refinement. It has been shown that these formats are particularly susceptible to fragmentation and aggregation, both in bulk solution and at interfaces.^11^ Moreover, the types of liabilities can vary significantly between different formats, and predicting them using computational tools remains challenging.^11^ These difficulties can delay their translation into successful therapeutics.

Key developability properties such as thermal stability, stability against aggregation, chemical stability, and viscosity must be optimized simultaneously, resulting in a complex multidimensional optimization challenge. Moreover, the design space is vast and complex, since the developability profile can be optimized by either protein engineering or formulation design. The latter pathway typically involves design guided by human expertise assisted by high-throughput (HTP) experimental screening.^12^ While expert-driven designs are commonly effective for conventional mAbs, the required experimental screening effort can be higher for alternative formats, where less historical information is available. Although commonly used design of experiments (DOE) methods provide statistical frameworks to mitigate the experimental effort,^13,14^ they still encounter substantial limitations, such as difficulty in managing high-order complexities and a tendency to become trapped in local optima.^15^

In response to these demands, machine learning (ML) coupled with (HTP-) screening has emerged as a powerful tool to systematically explore the vast formulation design space and optimize multiple biophysical properties simultaneously while drastically reducing the number of required experiments.^16–20^ Especially, Bayesian Optimization (BO), a sequential ML technique for the global optimization of black-box functions, has shown great potential.^17,21^ By utilizing a probabilistic surrogate model that approximates the system’s behavior, the BO algorithm iteratively proposes new experiments based on a defined acquisition function, balancing exploitation and exploration of the design space. BO is a highly sample-efficient global optimization technique that can accommodate observation noise and propose batches of new observations, making it especially well-suited for experimental screening.

We have previously demonstrated the effectiveness of BO in optimizing two biophysical properties of mAb fragments by identifying the ideal formulation composition with a limited number of experiments.^17^ Now, we demonstrate that BO can be applied to more complex optimization setups and serve as an effective starting point for identifying correlations between parameters and properties in relevant regions of the high-dimensional design space. To this aim, we first increased the multidimensionality of the problem by simultaneously optimizing three key biophysical properties—melting temperature *T*_m_, diffusion interaction parameter *k*_D_, and stability against air–water interfaces. We selected bococizumab-IgG1 as our model system, a mAb previously reported to have poor biophysical properties^6^ and later discontinued in clinical trials.^22^ Notably, although bococizumab was originally evaluated as an IgG2 subtype in clinical studies, we adopted the IgG1 normalized version from Jain et al.^6^ because more comprehensive developability data are available for this variant. Using our approach, we identified formulation compositions that simultaneously improved all three target properties in only 33 experiments. BO seamlessly integrates domain-specific constraints such as osmolality, pH, or concentration limits, enabling users to navigate within defined limits. We show that the data collected by our method also provide valuable insights into the effect of individual excipients on each biophysical property across formulations. Furthermore, our analysis highlights the need of balancing trade-offs between conflicting properties, such as the opposing effect of pH on *T*_m_ and *k*_D_—where high pH favors high *T*_m_, while low pH supports high *k*_D_.

## 2 Materials and Methods

### 2.1 Bayesian Optimization Algorithm

For the BO, we utilized the ProcessOptimizer package (version 0.9.4),^23,24^ which is built on scikit-optimize.^25^ The optimization targeted three objectives (thermal, colloidal, and interfacial stability) while considering six input variables. Instead of directly using the concentrations of each excipient as inputs for the algorithm, we explicitly defined pH as one of the variables, given its critical role in antibody formulations.^26^ This finally led to the input variables *c*_Sorbitol_, *c*_Arginine_, pH, *f*_Glu. acid_, *f*_Asp. acid_, and *f*_HCl_, with *f*_*x*_ being the relative fraction of acid *x*; the total acid concentration is implicitly given by the pH. The variables were normalized to a unit hypercube, and the objectives were standardized to have zero mean and unit variance. Each objective was modeled by an independent Gaussian process (GP) using a Matern 5/2 kernel, and the length scales and output variance were determined using maximum likelihood estimation. New points were suggested with a 75% probability via the exploitation route, where the Pareto front was generated based on the GPs and NSGA-II,^27^ using a generation size of 100 and a total of 100 generations. The ProcessOptimizer code was modified to allow for the incorporation of inequality constraints, more specifically to avoid the possibility that the sum of all acid fractions *f* exceeded unity and that the osmolality stayed within the specified range. Concentrations were reconstructed from the independent variables using the package pHcalc.^28^ If a genetic move led to a constraint violation, it was discarded and attempted again. Finally, the best Pareto point was selected based on its minimum distance to preexisting observations in objective and in variable space. These two were linearly combined, and the maximum value was chosen as the next experiment.^23^ The exploration route (*p* = 25%) works by minimizing the “Steinerberger sum”,^23^ a measure of proximity to explored points in the variable space, from 20 random starting points, with the mentioned constraints added. The best value was chosen for the next experiment. The batch size was set to five with a greedy strategy for suggesting multiple points (Kriging believer^29^), the initialization consisted of 13 points, randomly sampled from a uniform distribution across the variable space. Performing one iteration takes 56 min on an Intel Core i9-12900K processor, measured by running the method with all collected data points as input, providing an upper-limit estimate for computational costs. The majority of CPU time (>99%) is spent reconstructing acid concentrations from the independent variables to enforce the osmolality constraint. To analyze convergence, the hypervolume was calculated based on standardized objectives and using the Python package pygmo (version 2.19.6).^30^

### 2.2 Antibody Expression and Purification

Recombinant bococizumab-IgG1 was produced in Chinese Hamster Ovary (CHO) host cells lacking the endogenous glutamine synthetase (GS) gene. CHO cells were transfected with a bococizumab-IgG1 expression plasmid containing a GS selection marker, and cells were hereafter aliquoted into plates where they were subjected to selection using glutamine deprival together with MSX supplement in CD-CHO medium (10743029, Thermo Fisher Scientific). Stable CHO clones were obtained in plates after 2–4 weeks of selection, and they were hereafter expanded into suspension culture and screened for productivity. One selected the bococizumab-IgG1-expressing CHO clone was upscaled into fed-batch culture for further production of material.

Recombinant bococizumab-IgG1 was purified by protein-A resin MabSelect SuRe (Cytiva) with acidic elution followed by size exclusion chromatography Superdex200 column (Cytiva) into a resulting HBS (Hepes buffered saline, pH 7.4) formulation buffer. Protein integrity and purity was analyzed using an Ultra-Performance Liquid Chromatographic method setup on an Arc system (Waters) running a BEH SEC Column 200 Å, 1.7 column (Waters), and a running buffer composed of 25 mM sodium phosphate, 25 mM disodium phosphate pH 6.8, 300 mM NaCl, and 5% isopropanol. Molecular masses of the purified mAb batches were analyzed using electrospray ionization time-of-flight mass spectrometry on a BioAccord system connected with an UPLC Acquity Premier system (Waters) with a MassPREP Desalt (Waters) column using an A-buffer composed of MQ-H_2_O/0.1% formic acid and a B-buffer composed of acetonitrile/0.1% formic acid for step elution. To measure the final protein concentration, a Lunatic spectrophotometer (Unchained Laboratories) was used with theoretically calculated extinction coefficients.

### 2.3 Preparation of the Formulations and Buffer Exchange

The formulations were prepared using a fixed concentration of 10 mM l-histidine (≥99.5%, Sigma-Aldrich) and varying concentrations of d-sorbitol (≥98%, Sigma-Aldrich), l-arginine (≥99.5%, Sigma-Aldrich), l-aspartic acid (≥98%, Sigma-Aldrich), l-glutamic acid (≥99%, Sigma-Aldrich), glacial acetic acid (VWR Chemicals), and hydrochloric acid (37%, VWR Chemicals). The respective limits for each excipient/property, as well as commercial ranges, are shown in Table 1. To account for the direct relationship between acid concentration and pH, we selected acid fractions as the design parameter instead of using concentrations for aspartic acid, glutamic acid, acetic acid, and HCl. Using these fractions and the desired pH, the corresponding acid concentrations were calculated using the Python package pHcalc.^28^ All formulations were filtered by using a 0.45 μm Durapore PVDF Membrane filter (Merck) before further use. Buffer exchange was performed using prewetted centrifugal filter devices with a 10 kDa cutoff (Amicon Ultra, Merck). The filter units were centrifuged at 3803*g* for 6 min at 4 °C to concentrate the sample and remove the initial buffer. A total of five spin filtration steps were performed to achieve thorough buffer exchange, resulting in a theoretical dilution factor of 0.00027 for the initial buffer. To confirm that the structural stability of the mAb was preserved, dynamic light scattering (DLS) and nanodispersive scanning fluorimetry (NANODSF) measurements were performed before and after the spin filtration process. As shown in Figure S1, no detectable changes in hydrodynamic size or thermal stability were observed, indicating that the mAb remained structurally intact throughout the buffer exchange procedure. The final mAb concentration was determined by measuring the absorption at 280 nm using a Nanodrop Lite (Thermo Scientific) and a theoretically calculated extinction coefficient.

**Table 1:** Overview of the Variable and Constraint Design Space, Their Commercial Ranges, and the Rationale behind Their Usage^a^

excipient/property	design space	commercial range	rationale
histidine	10 mM (fixed)	3–50 mM	most common buffer in commercial mAb formulations
sorbitol	0–550 mM	88–270 mM	common excipient to increase osmolality and improve stability
arginine	0–250 mM	25–200 mM	common excipient to reduce aggregation and viscosity of high- concentration mAb formulations
frac. aspartic acid	0–1		alternatives for pH adjustment of mAb formulations
frac. glutamic acid	0–1		
frac. acetic acid	0–1		
frac. HCl	0–1		commonly used to adjust pH of mAb formulations
pH	4.5–7.5	4.8–8	
osmolality	100–600 mOsm kg^–1^	200–550 mOsm kg^–1^	tolerable range

a The commercial range refers to commercially available mAb formulations.^41,45,49–51^

### 2.4 Measurement of the Melting Temperature *T*_m_

The melting temperature *T*_m_ of the mAb in the different formulations was measured in triplicates via NANODSF using a Prometheus Panta (Nanotemper Technologies) and standard glass capillaries filled with 10 μL mAb solution at 7 mg mL^–1^. The intrinsic fluorescence of the mAb at 330 and 350 nm upon excitation at 280 nm was measured while heating the samples at a constant ramp of 1 °C min^–1^ from 25 to 90 °C. Data analysis was performed using NanoTemper’s inbuilt analysis software. *T*_m_ was derived from the maximum of the first derivative of the fluorescence ratio at 350 and 330 nm,^31^ which is associated with the unfolding of the C_H_2 domain.^32,33^ An exemplary unfolding curve is shown in Figure S2.

### 2.5 Measurement of the Diffusion Interaction Parameter *k*_D_

For each formulation, the mAb was diluted to a range of concentrations (2, 3.25, 4.5, 5.75, and 7 mg mL^–1^), and the diffusion coefficient *D* was measured in triplicates via DLS at 25 °C using a Prometheus Panta (Nanotemper Technologies) and standard glass capillaries filled with 10 μL mAb solution. The diffusion interaction parameter *k*_D_ was calculated based on the linear relationship of the diffusion coefficient *D* and protein concentration *c*, as given in the following equation^34^

1 $$D=D_{0}\left(1+k_{D}c\right)$$

where *D*_0_ is the diffusion coefficient at infinite dilution. Figure S3 presents representative plots of the diffusion coefficient at varying mAb concentrations for low, near-zero, and high *k*_D_ values, along with corresponding linear fits and their *R* ^2^ values. Higher absolute *k*_D_ values result in more accurate linear regressions, as indicated by *R* ^2^ values close to one.

### 2.6 Agitation Stress Assay

Solutions of mAb at 1 mg mL^–1^ in the respective buffer were prepared, and 150 μL of each sample was transferred into 1.5 mL screw neck vials (clear, 32 × 11.6 mm, BGB) and sealed with parafilm. Quadruplicates of each sample were placed horizontally on a shaking incubator (Labnet International), tightly secured using tape, and shaken at 1200 rpm for 6 d and 17.5 h. Immediately after shaking, the samples were transferred into microcentrifuge tubes and centrifuged for 1.5 h at 25 °C and 16,100*g* to separate the mAb monomers from the aggregates. The mAb concentration in the supernatant was determined by measuring the absorbance at 280 nm using a Nanodrop Lite (Thermo Scientific). The retained monomer (RM) after the agitation stress was calculated based on the following equation

2 $$RM_{Agi}\;\left[\%\right]=100\%-\left(\frac{c_{ref}-c_{shaken}}{c_{ref}}\times 100\%\right)$$

where *c*_ref_ and *c*_shaken_ are the concentrations of the unshaken reference and the shaken sample, respectively.

## 3 Results and Discussion

### 3.1 Sequential Experimental Design Using Bayesian Optimization

In this study, we simultaneously optimized three biophysical properties—thermal, colloidal, and interfacial stability—of the model mAb bococizumab-IgG1 by identifying the ideal formulation composition. Thermal stability was assessed by the first melting temperature *T*_m1_, which corresponds to the unfolding of the C_H_2 domain^32,33^ and signals the onset of destabilization. Colloidal stability was evaluated through the diffusion interaction parameter *k*_D_, which has proven to correlate to a certain extent with antibody self-association and viscosity at high concentration.^35–37^ A negative *k*_D_ indicates net attractive interactions, while a positive *k*_D_ indicates net repulsive self-interactions. Interfacial stability was measured by evaluating the RM fraction after exposing the protein solution to an agitation stress assay (see details in Materials and Methods Section 2.6).

Multidimensional optimization problems inherently involve trade-offs between the individual objectives as it is typically not feasible to find a single solution that optimizes all objectives simultaneously. Therefore, we applied the concept of Pareto optimality: a solution is Pareto optimal if no objective can be improved without negatively affecting another objective.^38^ Finding these Pareto optimal solutions while performing the lowest possible number of experiments is especially relevant for the pharmaceutical industry, where experiments are extremely time and resource intensive. An additional challenge is the high dimensionality of our design space, with a total of six parameters that can be tuned independently: sorbitol and arginine concentrations; fractions of aspartic acid, glutamic acid, acetic acid, and HCl; and pH. In order to navigate this design space efficiently, we employed BO, a highly efficient global optimization technique suitable for optimizing noisy and expensive-to-evaluate functions.^39^ Specifically, we utilized a modified version of the ProcessOptimizer package,^23^ built on scikit-optimize.^25^ The overall workflow is summarized in Figure 1. After initial experiments were conducted to quantify the three objectives for a given set of initial compositions, GP models are subsequently trained on each objective. To suggest the next experiment, the algorithm selects either an exploitation strategy with a probability of 75% or focuses on exploration of the design space with a probability of 25%. In the exploit case, the Pareto front is constructed using Gaussian surrogate models and the NSGA-II genetic algorithm.^27^ A new point is then selected based on its distance from previous observations in both the objective and feature spaces. For the explore route, the GPs are not utilized, and a new point is chosen solely on the basis of its distance from previous observations in the feature space. This approach further increases robustness by reducing the risk of getting trapped in local optima by preventing large uncovered regions in the design space. See Materials and Methods Section 2.1 for more details. In both cases, nonlinear constraints are applied to ensure pH and osmolality remain within the specified range. To enable the parallel execution of *n* experiments, a greedy strategy (Kriging Believer^29^) is applied, which involves retraining the model *n* – 1 times on fantasized results. This process ultimately yields a new set of experiments for the next optimization iteration. We monitored the hypervolume of the Pareto front to determine the convergence of the formulation optimization.

**Figure 1:**
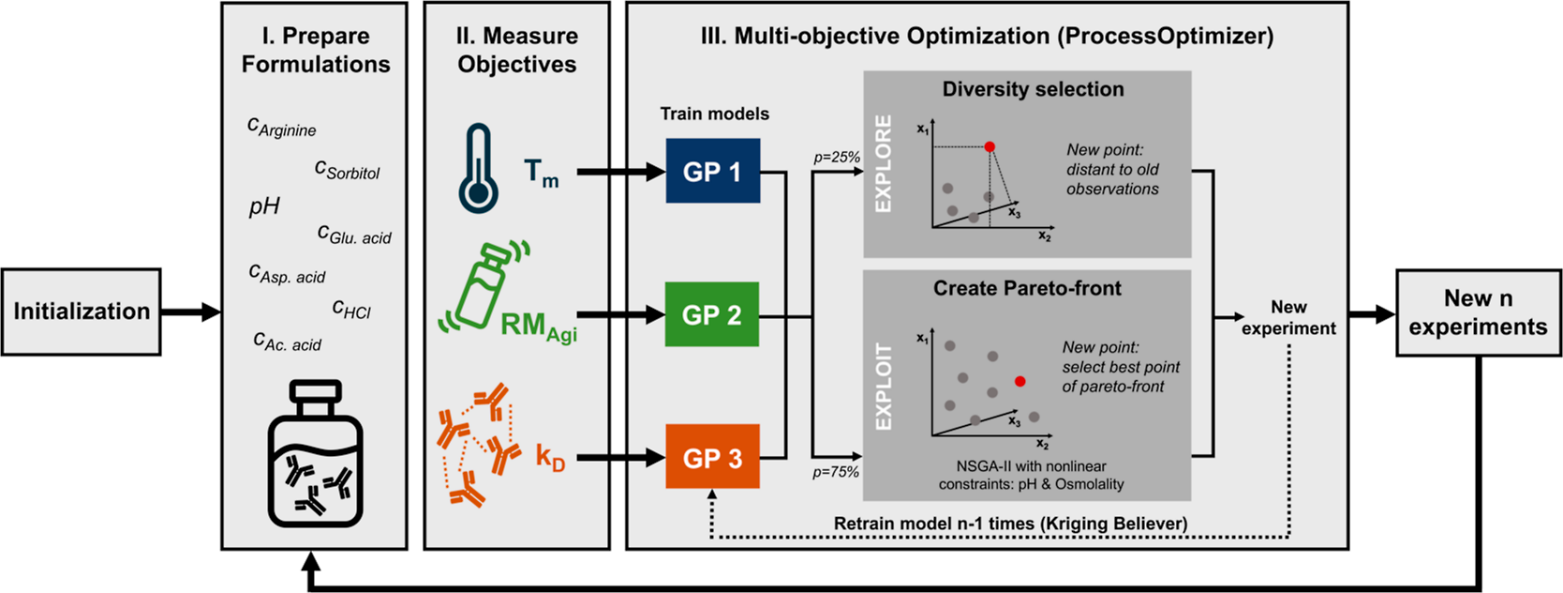
Schematic illustration of the sequential multiobjective optimization method. Initial experiments are conducted to quantify the three objectives (*T*_m_, *k*_D_, and RM_Agi_) and train GP models accordingly. The next experiment is proposed by selecting either an exploitation strategy that constructs a Pareto front based on GP models or an exploration strategy based solely on distance to previous observations. In both cases, nonlinear constraints are applied to ensure pH and osmolality remain within the specified range. To enable parallel execution of *n* experiments, a greedy Kriging Believer strategy retrains the model *n* – 1 times on fantasized results.

### 3.2 Accelerated Identification of Pareto Optimal Formulations

Most commercial mAb formulations include tonicity-modifying agents, sugar/polyol stabilizers, buffers, surfactants, amino acid stabilizers, viscosity modifiers, chelators, and antioxidants.^40^ In this study, we limited the formulation design space to sorbitol, arginine, aspartic acid, glutamic acid, acetic acid, HCl, and pH. The specific ranges for each excipient/property, along with commercial ranges and the rationale for their usage, are listed in Table 1. Arginine-HCl is among the most used excipients in commercial antibody products, while pH adjustment with aspartic acid, glutamic acid, and acetic acid instead of HCl is limited. As there are reported negative effects of HCl on the physical stability of proteins,^41^ and using other acids, such as glutamic acid, aspartic acid, and acetic acid, can contribute positively,^42–44^ we decided to also include these excipients as variables in the formulation design space. Additionally, a fixed concentration of 10 mM histidine was included in each formulation, given its prevalence as a buffer in modern mAb formulations.^45,46^ A key factor to consider in formulation design is osmolality, which should ideally match physiological levels of blood (275–295 mOsm kg^–1^ ^47^). Accordingly, we incorporated a constraint to keep osmolality within a tolerable range of 100–600 mOsm kg^–1^,^48^ which slightly broadens the design space compared to commercial high-concentration mAb therapeutics (200–550 mOsm kg^–1^).^49^

As shown in Figure 2A, our optimization led us to identify near Pareto optimal formulations by performing only 33 experiments, a reduction of several orders of magnitude compared to a full screen of the design space. Even when compared to common DOE methods such as Central Composite Design^52^ and Box-Behnken Design^53^ (see Table S1 for details), our approach required less than half the number of experiments. In high-dimensional spaces, BO is particularly advantageous because it optimizes the sample selection process instead of exhaustively searching the entire design space, as nonadaptive DOE methods do. Moreover, while BO efficiently identifies near global optima, DOE methods are more prone to get trapped in local optima and may fail to capture complex behavior, often requiring additional experimentation. Although BO still cannot guarantee the true global optimum and its search retains a stochastic component, its exploration–exploitation balance reduces the chance of becoming stuck in local optima created by rugged response surfaces and stochasticity, such as experimental noise.

**Figure 2:**
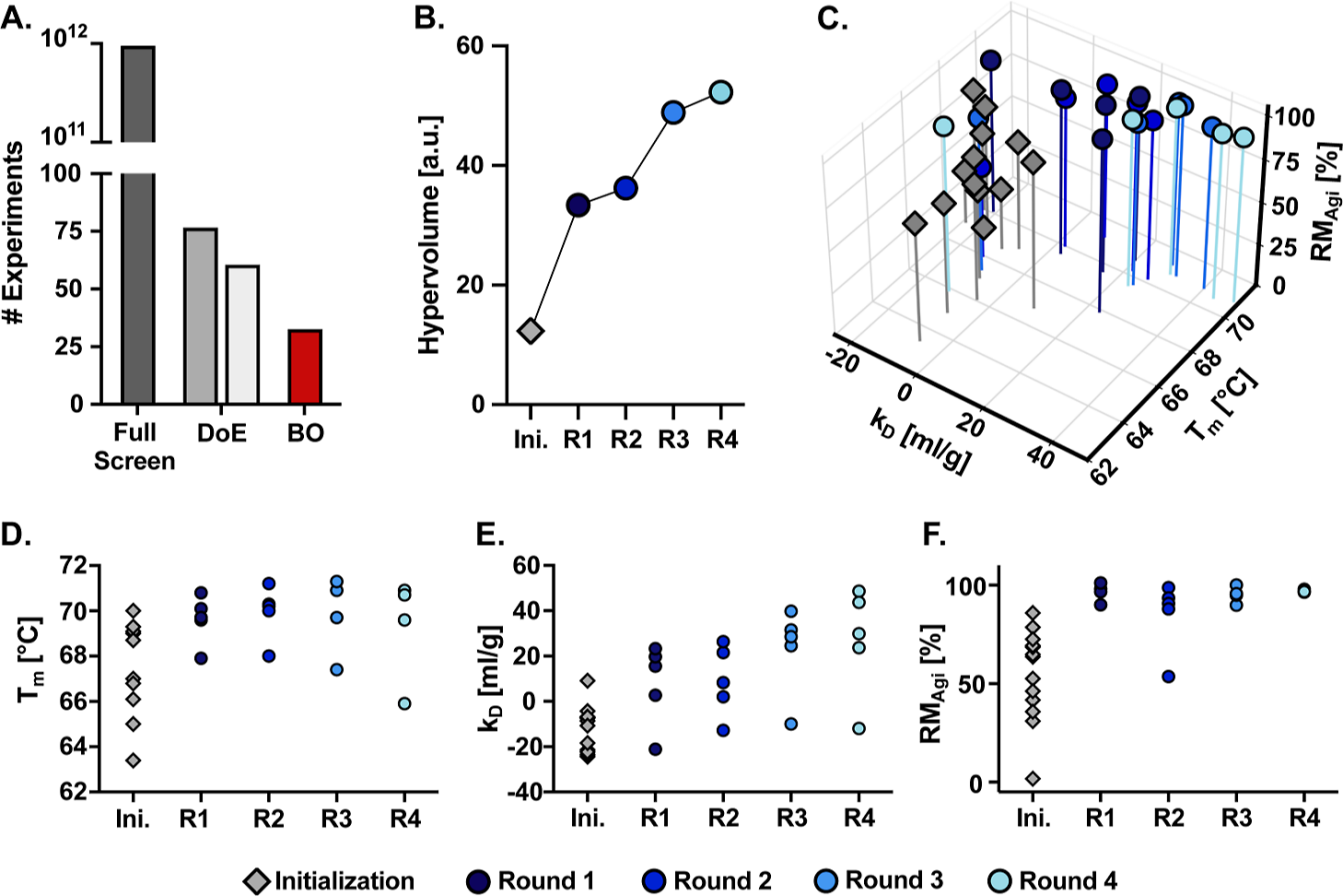
(A) Comparison between the number of experiments required for a full screen, DOE, and BO approach. For the DOE approach, two common methods are given as examples, both based on a quadratic response surface: Central-Composite-Design^52^ (left) and Box-Behnken-Design^53^ (right). (B) Evolution of the hypervolume over multiple iterations. (C) Simultaneous improvement of the three biophysical properties (melting temperature *T*_m_, diffusion interaction parameter *k*_D_, and retained monomer fraction after agitation stress assay RM_Agi_). (D–F) Progression of the biophysical properties *T*_m_, *k*_D_, and RM_Agi_ over multiple iterations.

To evaluate the performance of our sequential BO method, we calculated the hypervolume at each optimization iteration. The hypervolume quantifies the size of the dominated region in the objective space, with higher values indicating better performance.^54^ As shown in Figure 2B, the hypervolume consistently increased across all optimization iterations, highlighting the effectiveness of our approach. We stopped after four iterations of optimization since the results suggested that the algorithm was nearing convergence.

Figure 2C displays a summary of all three biophysical properties of mAb in each formulation. The corresponding values with their 95% confidence intervals are provided in Table S2, and the formulation compositions are in Table S3. Additionally, Figure S4 reports the per-formulation hypervolume, calculated individually for each formulation, indicating its performance across all objectives relative to the reference point. The evolution of each individual property over the optimization iterations is shown in Figure 2D–F for *T*_m_, *k*_D_, and RM_Agi_, respectively. After each iteration, the algorithm identified progressively improved formulations, effectively balancing trade-offs between the individual properties. Notably, the algorithm identified formulations with substantially enhanced properties compared to the initial set, particularly for *k*_D_, which improved from 9.1 mL g^–1^ in the best initial formulation to 48.6 mL g^–1^ in the final optimization iteration. This greatly exceeds the 20 mL g^–1^ benchmark proposed by Kingsbury et al.^37^ to distinguish between well- and poorly behaved mAbs. The melting temperature was improved from 70.0 °C for the best initialization formulation to the highest value of 71.3 °C after the third optimization iteration. All melting temperatures beyond the initialization iteration exceed the 64.2 °C threshold reported by Jain et al.^55^ in their analysis of mAbs, which were approved until 2022. RM_Agi_ rapidly converged, maintaining values between 90% and 100% for almost all formulations following the initialization. The results for *T*_m_ and *k*_D_ clearly demonstrate the balance between exploitation and exploration in the BO method. The algorithm selected the exploration route with a 25% probability and the exploitation route with a 75% probability, resulting in approximately four formulations based on exploitation and one based on exploration per iteration. In all optimization iterations, *T*_m_ and *k*_D_ measurements for four of the five formulations clustered tightly together, reflecting the exploitation strategy, while one formulation displayed lower values, most likely representing an exploration point.

Optimization progress in the last three iterations appears to be driven mainly by improvements in *k*_D_ while maintaining high *T*_m_ and RM_Agi_, as shown by the nearly constant Pareto front in the *T*_m_-RM_Agi_ space (Figure S5) over this period. We also assessed how well the GP models predicted the outcomes of subsequent iterations using the mean absolute error (MAE) as the metric (Figure S6). For all three objectives, prediction quality improved after the first iteration as more training data became available. For *k*_D_, however, the prediction error rose again in the final two iterations, aligning with the comparatively slower convergence of this objective.

### 3.3 Effects of Individual Excipients on Biophysical Properties

In addition to identifying Pareto optimal formulations, the combined BO and experimental screening approach yields a data set that spans the relevant regions of the design space. A principal component analysis (PCA) plot showing the convergence of the formulations within the design space is provided in Figure S7. This collected data set provides a strong foundation for analyzing the impact of individual variables under optimized conditions. For this purpose, we first analyzed the evolution of the three key design parameters—pH, sorbitol, and arginine concentration—over the initialization and four optimization iterations, as illustrated in Figure 3A–C. While the formulations of the initialization exploit the entire pH design space (4.5–7.5), the pH settles down to an average value of approximately 6 ± 0.5 as the optimization progresses. This is in line with modern commercially available mAb formulations, which have a slightly acidic average pH of 5.8.^51^ The sorbitol concentration remains relatively dispersed throughout the optimization process but tends to center around 300 ± 100 mM as optimization progresses. Arginine concentration follows a clear trend: with the exception of one formulation per optimization iteration, likely reflecting the exploration route of our algorithm, all formulations exhibit negligible arginine content.

**Figure 3:**
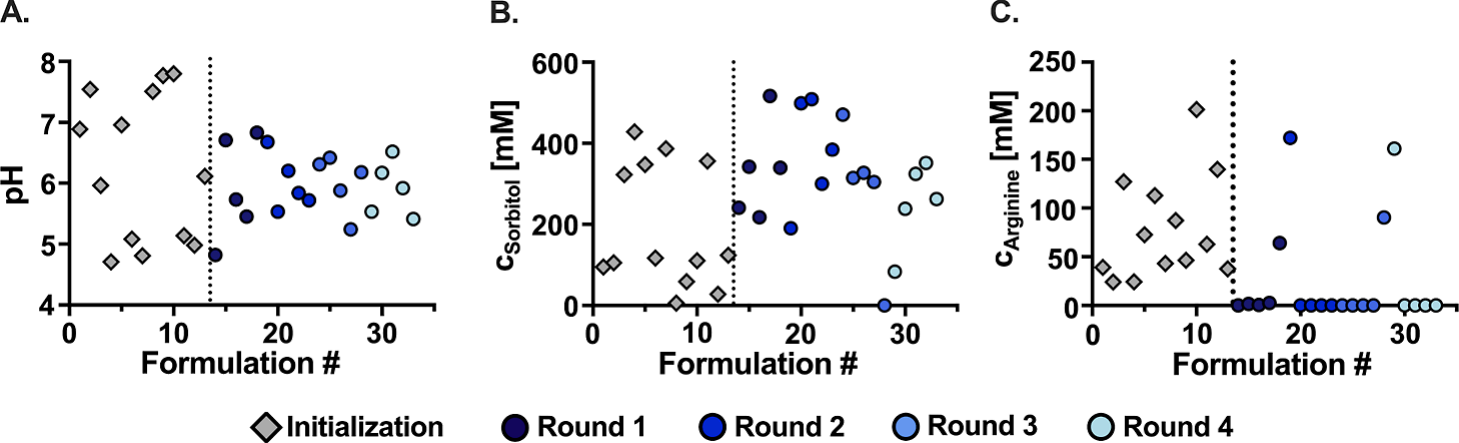
Evolution of the (A) pH and (B) sorbitol and (C) arginine concentration over the initialization and four optimization iterations.

To elucidate the factors driving the evolution of the key design parameters, we analyzed their individual impact on the biophysical properties (Figure 4A–C). Our analysis focused on *T*_m_ and *k*_D_ since RM_Agi_ rapidly converged and remained between 90% and 100% for nearly all formulations after initialization. Figure S8 further explores the influence of key design parameters by presenting the per-formulation hypervolume as a function of each parameter, identifying conditions that yield the most favorable trade-offs across all objectives. Additionally, Figure S9 displays the measurement results of all objectives against the individual key design parameters. Notably, interactions among formulation components can induce nonlinear effects on the biophysical properties, which the BO algorithm effectively counts for.

**Figure 4:**
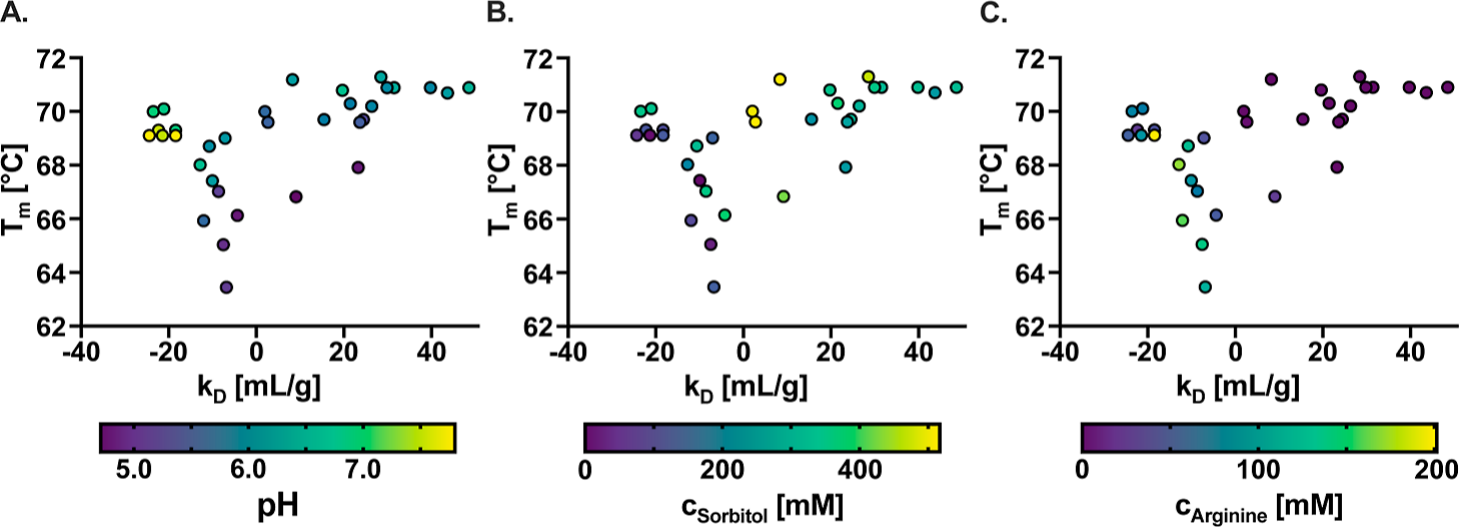
Individual effects of (A) pH and (B) sorbitol concentration and (C) arginine concentration on the melting temperature *T*_m_ and the diffusion interaction parameter *k*_D_.

A clear trade-off emerged when assessing the influence of pH on *T*_m_ and *k*_D_: high pH values favored increased *T*_m_, while lower pH levels promoted higher *k*_D_ values. This can be rationalized by considering that low pH values—significantly diverging from the isoelectric point (PI) of bococizumab-IgG1 (8.76 ^56^)—amplify intermolecular electrostatic repulsion, thereby increasing *k*_D_ and enhancing colloidal stability. Conversely, high pH levels closer to the protein’s PI enhance conformational stability and thus increase *T*_m_. As the pH moves away from the PI, the protein’s net charge increases, causing stronger intramolecular electrostatic repulsion, which in turn destabilizes the protein.^57^

The attempt to explore the application of aspartic acid, glutamic acid, and acetic acid instead of HCl for pH adjustment of arginine formulations did not provide any strong conclusions. This is likely the consequence of the arginine concentration being reduced significantly in formulations prepared in iterations two, three, and four, leading to the simultaneous reduction in added levels of acids. Furthermore, we included all four acids as combinatorial variables, making it more difficult to derive clear effects from the individual acids from the limited number of formulations tested. Based on Spearman correlations (see Figure S10), we observe the indication of a positive effect of fraction aspartic acid on all readouts, while acetic acid gives a negative effect (ranking: aspartic acid > glutamic acid > HCl > acetic acid); however, based on limited data, this requires further testing in future studies to derive any conclusions.

The addition of sorbitol up to an intermediate concentration of approximately 300 mM had a positive impact on all of the objectives, as shown in Figures 4B and S9. As illustrated in Figure S8, the highest per-formulation hypervolume was achieved at a sorbitol concentration of 324 mM. For *T*_m_, the relationship appears to be generally monotonic, with values starting to plateau at intermediate sorbitol concentrations. The maximum *T*_m_ was observed at 471 mM sorbitol—significantly exceeding the commercially available limit of 270 mM (see Table 1). In contrast, the maximum *k*_D_ value was reached at an intermediate sorbitol concentration of 324 mM, while higher concentrations resulted in reduced *k*_D_ values. However, further experiments are required to confirm the observed decline in *k*_D_ at sorbitol concentrations above 300 mM, given the limited number of data points, relative to the complexity of the formulation design space. The stabilizing effects of polyols and sugars, such as sorbitol, have been well documented since the 1980s,^58^ leading to their widespread use as excipients in drug formulations.^45^ The proposed mechanism involves their preferential exclusion from the protein surface, promoting a more compact and thermodynamically stable structure.^58,59^ Interestingly, this mechanism has recently been shown across the human proteome, showing this osmolyte function also in a complex biological matrix.^60^

As shown in Figures 4C and S9, the addition of arginine reduced *T*_m_ and, more significantly, *k*_D_ values. Formulations containing arginine in the optimization iterations consistently resulted in unfavorable *k*_D_ values, ranging from −21 to −10 mL g^–1^. This trend is further supported by Figure S8, which demonstrates that the highest per-formulation hypervolumes are associated with an arginine concentration of 0 mM. Consistently, Figure S11 reports a Spearman correlation coefficient of −0.94 for *k*_D_, underscoring the strong negative relationship between arginine concentration and colloidal stability. This negative effect was quickly identified by the BO algorithm, leading to the exclusion of arginine in nearly all formulations after the initialization phase. Several studies have documented the negative effects of arginine, which appear to be highly system-dependent and remain poorly understood.^41,61–64^ A possible explanation for the reduced *k*_D_ is that arginine, positively charged at formulation-relevant pH levels, increases the ionic strength of the solution, thereby reducing repulsive intermolecular interactions and lowering colloidal stability.^61^ The reduction in *T*_m_ could be linked to the chaotropic properties of arginine’s guanidinium group, which may disrupt the tertiary structure of the mAb, leading to decreased conformational stability.^63^ This system-dependent effect of arginine underscores the advantages of ML approaches in accelerating the design of tailored formulations based on protein identity.

### 3.4 Correlation with High Protein Concentration Measurements

We then investigated the relationship between *k*_D_ and other key developability properties at high mAb concentrations (125 mg mL^–1^), including viscosity, opalescence, and oligomerization measured by small-angle X-ray scattering (SAXS) (measurement details in Supporting Information). To comprehensively capture the objective space, we selected nine formulations—encompassing Pareto optimal formulations as well as those with high, intermediate, and low *k*_D_ and *T*_m_ values. At high mAb concentrations, pH appears to play a critical role in viscosity and opalescence: formulations at lower pH exhibit reduced viscosity and opalescence compared to those at higher pH (Figure S12C). This trend appears to be a general characteristic of high-concentration mAb formulations.^65^

Contrary to recent literature,^35,37,66^ we observed no significant correlations between *k*_D_ and properties at high mAb concentrations (see Figure S12 and the data in Tables S4 and S5). Notably, the aforementioned studies compared different variants within fixed formulations, demonstrating that *k*_D_ can effectively rank molecules under those conditions. However, when different formulations are compared as in our study, the utility of *k*_D_ as a predictor for high-concentration behavior seems more complex. These findings underscore that extrapolating results from low to high protein concentrations should be approached with caution, as no single property can reliably serve as a proxy for the others. Consequently, the dimensionality and complexity of the optimization space increase, requiring simultaneous optimization of multiple properties that are interconnected but not proportionally linked. Furthermore, assays designed to measure properties at high protein concentrations currently require large quantities of material. Collectively, these challenges highlight the value of approaches such as BO in minimizing the number of experiments required for multidimensional optimization.^17,18^ Combined with advances in low-volume experimental characterization techniques—such as microfluidics^67,68^ —this strategy has the potential to accelerate developability and formulation studies while reducing associated costs.

## 4 Conclusion

Innovative formulation design strategies are becoming increasingly essential as emerging engineered antibody formats often exhibit poor developability profiles.^11^ The rising demand for subcutaneous drug delivery—often requiring protein concentrations above 150 mg mL^–1^—introduces additional challenges, particularly elevated viscosity and protein aggregation driven by short-range interactions.^69^ While computational methods exist to assess the influence of excipients on biophysical properties,^70–72^ their predictive power remains limited, especially for novel antibody constructs that lack historical expertise and well-established databases comparable to those available for conventional mAbs. Advancing these models requires systematic experimental data to uncover nontrivial effects, a process that ML-driven methods can significantly enhance by integrating data-driven insights.^17^

Here, we demonstrated that combining BO with experimental screening is an effective method for identifying formulation conditions for multiple optimization targets. We simultaneously improved three key biophysical properties: melting temperature *T*_m_, diffusion interaction parameter *k*_D_, and RM following protein agitation. By modeling each biophysical property with a separate GP, our method allows for the seamless integration of additional properties at later stages while leveraging prior knowledge, making it highly versatile. Notably, our optimization required only 33 experiments—several orders of magnitude fewer than a full screen and only half as many as the simplest DOE approach. Unlike traditional DOE methods, our BO approach effectively manages complex interactions among excipients, while its exploratory component minimizes the risk of being trapped in local optima. Moreover, the data collected with our method provide an excellent starting point for gaining deeper insights into the specific effects of individual excipients on biophysical properties across formulations. For example, the BO algorithm rapidly identified the adverse effects of arginine on all biophysical properties, which resulted in its removal from almost all formulations following initialization. While the mechanisms behind arginine’s impact remain poorly understood and highly system-dependent, this case highlights the significant advantages of ML strategies in designing tailored protein-specific formulations.

## Supplementary Material



## Notes

Additional experimental details, materials, and methods, data analyses, detailed measurement results, and formulation compositions (PDF)
