# Supplementary material for: Bayesian Optimization for Efficient Multiobjective Formulation Development of Biologics
Source: Mol Pharm. 2025 Sep 26;22(11):6636–45. doi: 10.1021/acs.molpharmaceut.5c00591 (PMC12587402; doi:10.1021/acs.molpharmaceut.5c00591)
Supplement: Supplementary file 1 [file mp5c00591_si_001.pdf]

Supporting Information

Bayesian Optimization for Efficient Multiobjective Formulation Development of Biologics

Isabel Waibel[1], Timo N. Schneider[1], Fiona J. Fischer[1], Poonpat Dumnoenchanvanit[1], Alina Kulakova[2], Tin Duy Nguyen[2], Thomas Egebjerg[2], Søren Bertelsen[3], Nikolai Lorenzen[2*], Paolo Arosio[1*]

[1] ETH Zürich, Department of Chemistry and Applied Biosciences,
Institute for Chemical and Bioengineering,
Vladimir-Prelog-Weg 1-5/10, 8093 Zürich, Switzerland

[2] Novo Nordisk A/S,
Therapeutics Discovery,
Novo Nordisk Park, 2760 Måløv, Denmark

[3] Novo Nordisk A/S,
Digital Science & Innovation,
Novo Nordisk Park, 2760 Måløv, Denmark

[*] Correspondence to: Paolo Arosio (paolo.arosio@chem.ethz.ch) and Nikolai Lorenzen (nlz@novonordisk.com)

Table S1: Comparison between a full screen and two common DoE methods (Box-Behnken and Central-Composite Design). k denotes the factors, which are in our study the pH and 5 parameters for the excipients. The levels of the factors, represented by n, were set to 100.

Design Method	Formula	No. of Experiments	Response Surface
Full Screen	n^k	10^{12}	No assumption
Box-Behnken Design	$2k(k-1)+1$	61	Quadratic
Central-Composite Design	2^k+2k+1	77	Quadratic

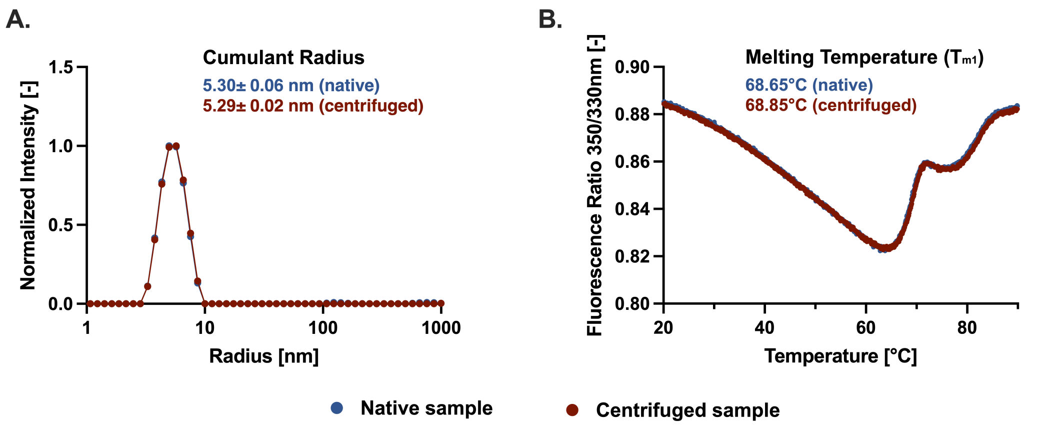

Figure S1: A. Normalized intensity DLS measurements and **B.** Thermal denaturation profile of Bococizumab-IgG1 (1 mg mL^{-1}, 20mM HEPES 150mM NaCl pH 7.4, three replicates per sample) before and after spin filtration. No detectable changes in hydrodynamic size or thermal stability were observed, indicating that the mAb remained structurally intact throughout the buffer exchange procedure.

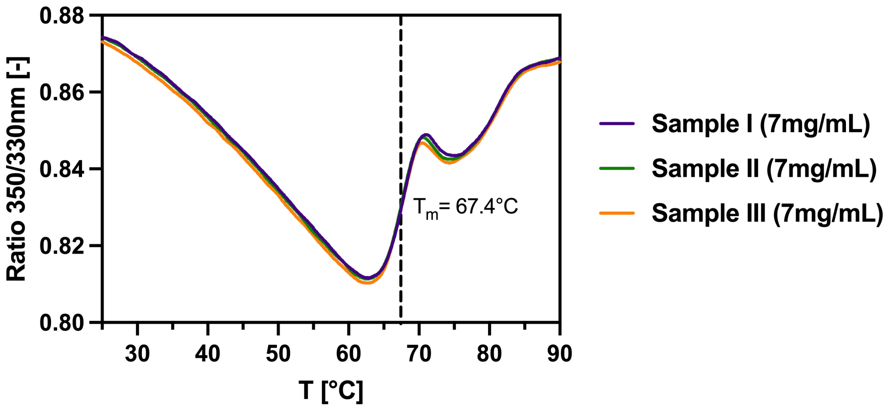

Figure S2: Representative thermal denaturation profile of Bococizumab-IgG1 (7 mg mL^{-1}, formulation 28, three replicates per sample) assessed by the intrinsic fluorescence ratio (350/330 nm). We considered the initial inflection point (marked with a dashed line in the graph), which can be assigned to the C_H2 domain unfolding, as the melting temperature T_m since it indicates the onset of structural changes.

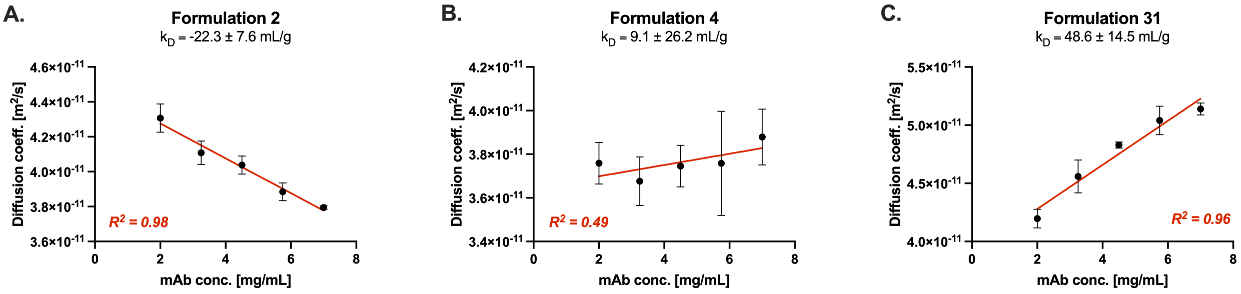

Figure S3: A.-C. Diffusion coefficients at varying mAb concentrations (2-7 mg mL^{-1}) with linear fits used to calculate k_D values. Formulations 2 (**A**) and 31 (**C**) are shown as examples of low and high k_D values, respectively, both yielding strong linear fits with high R^2 values. Formulation 4 (**B**) represents a case with a k_D value near zero, resulting in a lower R^2 value.

Table S2: All measurement results with their respective 95% confidence interval.

Formulation	T_m [° C]	k_D [mL/g]	RM_{Agi} [%]
1	69.3 ± 0.1	−18.4 ± 4.9	72.6 ± 15.5
2	69.3 ± 0.1	−22.3 ± 7.6	78.6 ± 7.0
3	68.7 ± 0.1	−10.7 ± 9.5	35.8 ± 23.0
4	66.8 ± 0.2	9.1 ± 26.2	85.9 ± 3.4
5	70.0 ± 0.0	−23.5 ± 1.6	46.2 ± 8.1
6	63.4 ± 0.1	−6.9 ± 2.8	69.3 ± 2.1
7	66.1 ± 0.1	−4.3 ± 3.5	68.6 ± 7.2
8	69.1 ± 0.1	−21.4 ± 3.3	41.7 ± 17.5
9	69.1 ± 0.1	−24.4 ± 1.6	31.0 ± 8.6
10	69.1 ± 0.0	−18.4 ± 3.3	1.8 ± 1.6
11	67.0 ± 0.1	−8.6 ± 4.6	52.5 ± 9.7
12	65.0 ± 0.1	−7.5 ± 2.7	64.8 ± 14.6
13	69.0 ± 0.1	−7.1 ± 4.5	63.7 ± 18.5
14	67.9 ± 0.2	23.3 ± 3.4	101.1 ± 2.7
15	70.8 ± 0.1	19.8 ± 4.7	96.6 ± 3.3
16	69.7 ± 0.1	15.5 ± 3.4	98.1 ± 1.4
17	69.6 ± 0.0	2.7 ± 4.5	96.6 ± 2.6
18	70.1 ± 0.1	−21.1 ± 4.0	90.0 ± 1.5
19	68.0 ± 0.2	−12.8 ± 3.2	53.6 ± 21.9
20	70.0 ± 0.1	2.0 ± 5.9	87.9 ± 1.3
21	71.2 ± 0.1	8.3 ± 5.6	90.7 ± 3.2
22	70.2 ± 0.0	26.3 ± 5.1	93.5 ± 2.1
23	70.3 ± 0.0	21.5 ± 1.3	98.7 ± 1.5
24	71.3 ± 0.0	28.5 ± 18.9	95.7 ± 1.4
25	70.9 ± 0.1	31.5 ± 20.3	100.1 ± 2.9
26	70.9 ± 0.2	39.8 ± 8.9	94.9 ± 3.1
27	69.7 ± 0.1	24.5 ± 5.7	95.2 ± 2.0
28	67.4 ± 0.2	−10.0 ± 2.4	89.8 ± 1.6
29	65.9 ± 0.1	−12.0 ± 11.1	96.7 ± 1.4
30	70.7 ± 0.1	43.7 ± 12.7	96.7 ± 4.6
31	70.9 ± 0.0	48.6 ± 14.5	96.4 ± 1.7
32	70.9 ± 0.0	29.9 ± 12.6	97.9 ± 2.1
33	69.6 ± 0.2	23.7 ± 7.4	97.6 ± 1.1

Calculation of Confidence Intervals

The 95% confidence intervals (CI) for the objectives T_m and RM_{Agi} were calculated based on **Equation 3**.

$$CI_{95\%} = \overline{x} \pm t_{(1-\frac{\alpha}{2};n-1)} * \frac{s}{\sqrt{n}} \tag{3}$$

$\overline{x}$ represents the mean of the estimate, $t_{(1-\frac{\alpha}{2};n-1)}$ the critical value of the t-distribution, s the sample standard deviation, and n the sample size.

Since the k_D value was determined based on a linear regression, the corresponding $CI_{95\%}$ was calculated based on:

$$CI_{95\%} = \overline{x} \pm t_{(1-\frac{\alpha}{2};n)} * SE_{\mathrm{kD}} \tag{4}$$

$\overline{x}$ represents the mean of the estimate, $t_{(1-\frac{\alpha}{2};n)}$ the critical value of the t-distribution, and SE_{kD} the standard error of k_D, which was calculated based on Gaussian error propagation:

$$SE_{kD}^2 = \left(\frac{\partial k_D}{\partial m}\right)^2 \cdot SE_m^2 + \left(\frac{\partial k_D}{\partial c}\right)^2 \cdot SE_c^2 \tag{5}$$

Equation 5 can be simplified to **Equation 7** by using the definition of k_D:

$$k_D = \frac{m}{c} \tag{6}$$

$$SE_{kD} = \sqrt{\left(\frac{SE_m^2}{c^2}\right) + \left(\frac{m^2 \cdot SE_c^2}{c^4}\right)} \tag{7}$$

Where m represents the slope and c the y-intercept of the linear regression.

Average 95% confidence intervals.

Objective	Critical value of t-distribution	Average $CI_{95\%}$
T_m [°C]	2.920	$\overline{X} \pm 0.1$ °C
RM_{Agi} [%]	2.353	$\overline{X} \pm 6\%$
k_D [mL g^{-1}]	3.182	$\overline{X} \pm 7.0$ mL g^{-1}

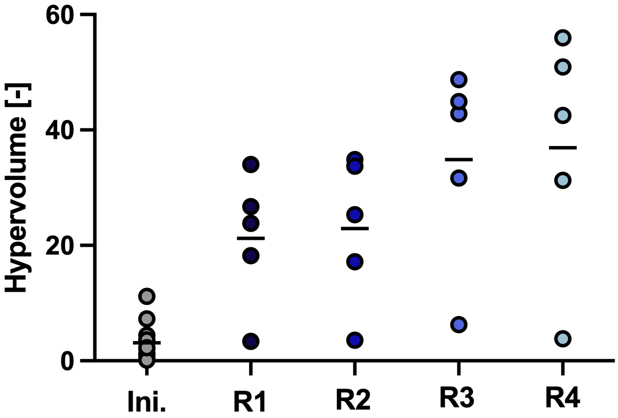

Figure S4: Per-formulation hypervolume, calculated individually for each formulation, indicating its performance across all objectives relative to the reference point. The horizontal line indicates the average value within each round. Across optimization rounds, both the average and the best-performing formulations improved, while each round consistently included one lower-performing formulation.

Table S3: Composition of the formulations.

Form.	pH	Histidine [mM]	Sorbitol [mM]	Arginine [mM]	Aspartic acid [mM]	Glutamic acid [mM]	Acetic acid [mM]	HCl [mM]
1	6.9	10	95	39	5	32	3	3
2	7.5	10	105	24	9	7	8	8
3	6.0	10	322	127	1	39	97	20
4	4.7	10	428	24	11	21	11	4
5	7.0	10	347	73	9	11	53	3
6	5.1	10	117	113	48	11	88	32
7	4.8	10	387	43	6	20	41	13
8	7.5	10	6	87	27	34	24	7
9	7.8	10	59	46	6	9	32	28
10	7.8	10	110	201	51	45	100	86
11	5.1	10	356	63	4	25	44	11
12	5.0	10	28	140	55	76	20	15
13	6.1	10	124	38	24	4	15	1
14	4.8	10	241	0	8	0	1	2
15	6.7	10	342	2	2	2	0	0
16	5.7	10	217	1	2	3	1	1
17	5.5	10	517	3	3	7	0	0
18	6.8	10	339	64	42	9	15	1
19	6.7	10	190	172	0	1	52	121
20	5.5	10	499	0	5	2	0	1
21	6.2	10	509	0	2	2	0	0
22	5.8	10	300	0	4	2	1	0
23	5.7	10	384	0	4	2	0	1
24	6.3	10	471	0	1	1	0	0
25	6.4	10	314	0	1	1	0	0
26	5.9	10	327	0	2	3	0	0
27	5.2	10	304	0	5	3	1	1
28	6.2	10	0	90	0	0	56	40
29	6.5	10	83	161	5	72	0	95
30	6.2	10	238	0	2	0	0	1
31	6.5	10	324	0	1	1	0	1
32	5.9	10	351	0	1	3	1	0
33	5.4	10	262	0	5	0	0	4

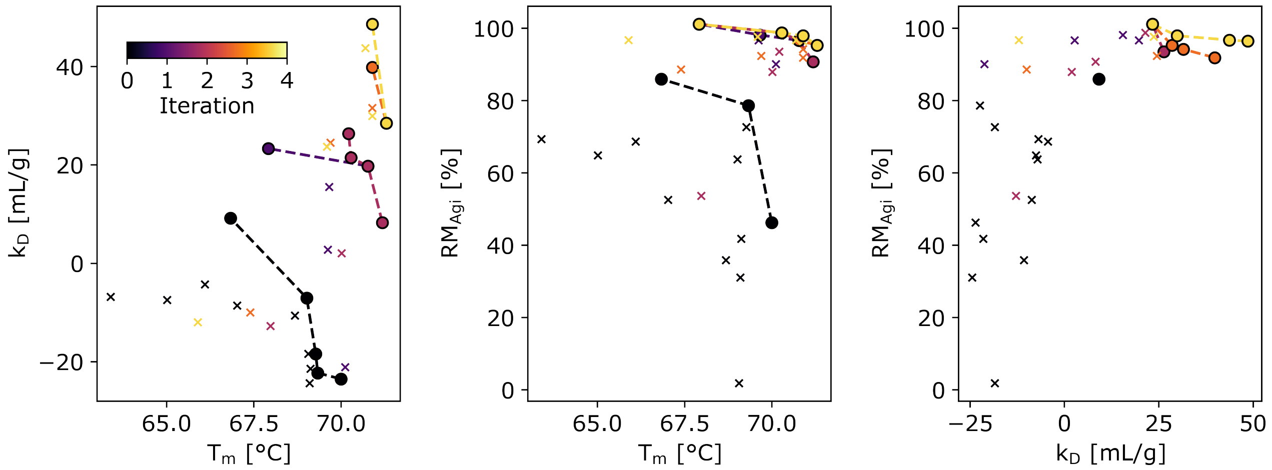

Figure S5: Projection of the optimization into 2-D space with corresponding Pareto fronts. Optimization progress in the last three iterations appears to be driven mainly by improvements in k_D while maintaining high T_m and RM_{Agi}, as shown by the nearly constant Pareto front in T_m-RM_{Agi} space (middle panel) over this period.

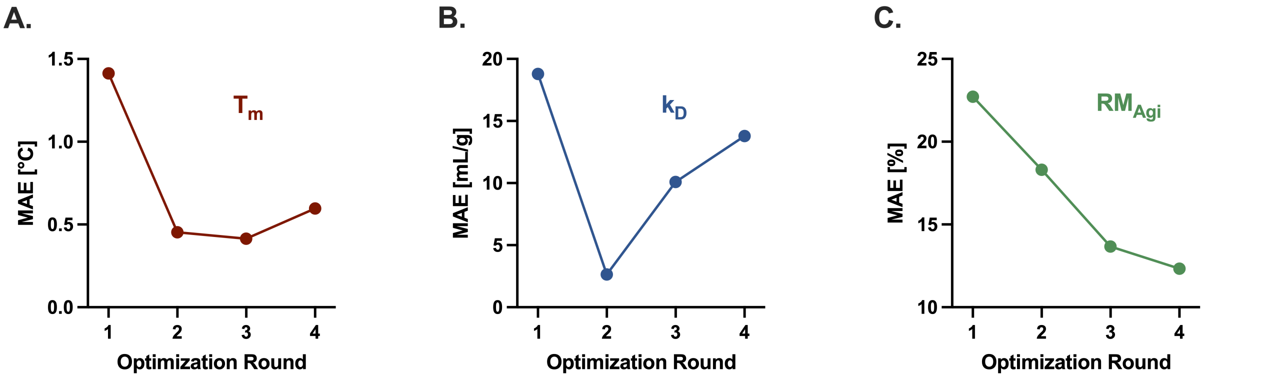

Figure S6: Mean absolute error of Gaussian process predictions for **A.** T_m **B.** k_D and **C.** RM_{Agi}, evaluated at each iteration using the data available up to that iteration for model training. For all three objectives, prediction quality improved after the first iteration as more training data became available. For k_D, however, the prediction error rose again in the final two iterations, aligning with the comparatively slower convergence of this objective.

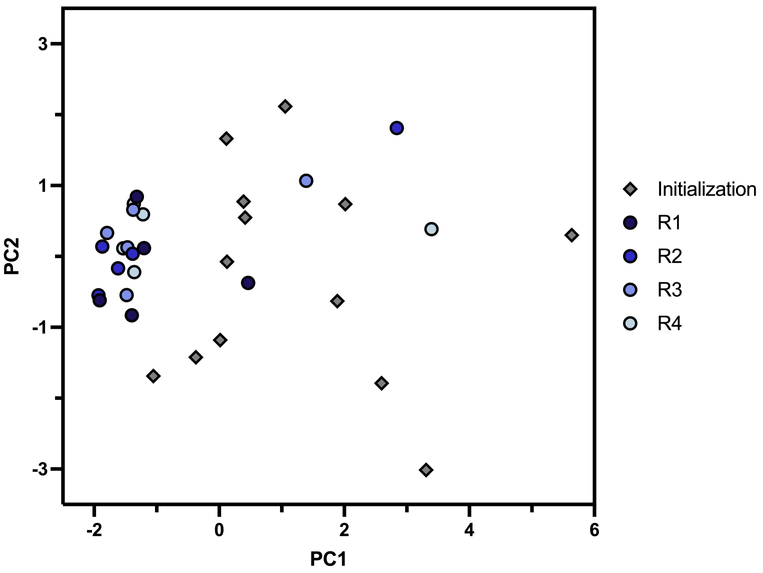

Figure S7: Principal component analysis (PCA) of standardized formulation parameters (Table S3) across the performed iterations. Most formulations proposed during the iterations are localized in a rather small region of the design space and are therefore more similar to each other than the initialization experiments.

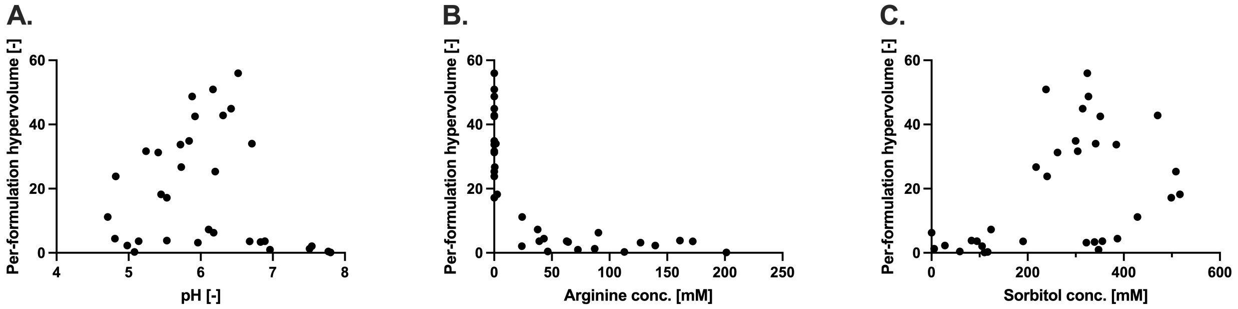

Figure S8: Per-formulation hypervolume vs. **A.** pH **B.** Arginine concentration **C.** Sorbitol concentration. The highest per-formulation hypervolumes (best trade-offs) are associated with an intermediate pH of 6.52, 0mM arginine, and an intermediate sorbitol concentration of 324mM.

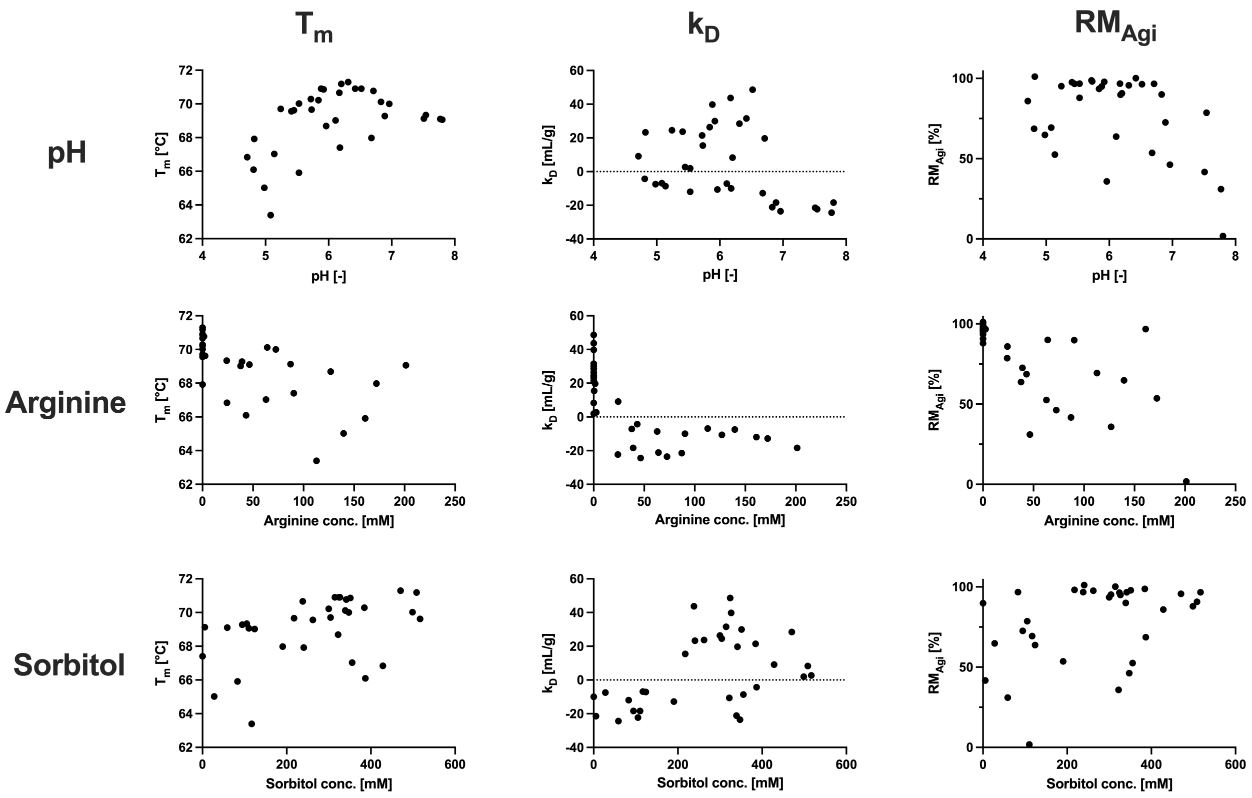

Figure S9: Values of different objectives plotted against individual main design parameters (T_m, k_D, and RM_{Agi}).

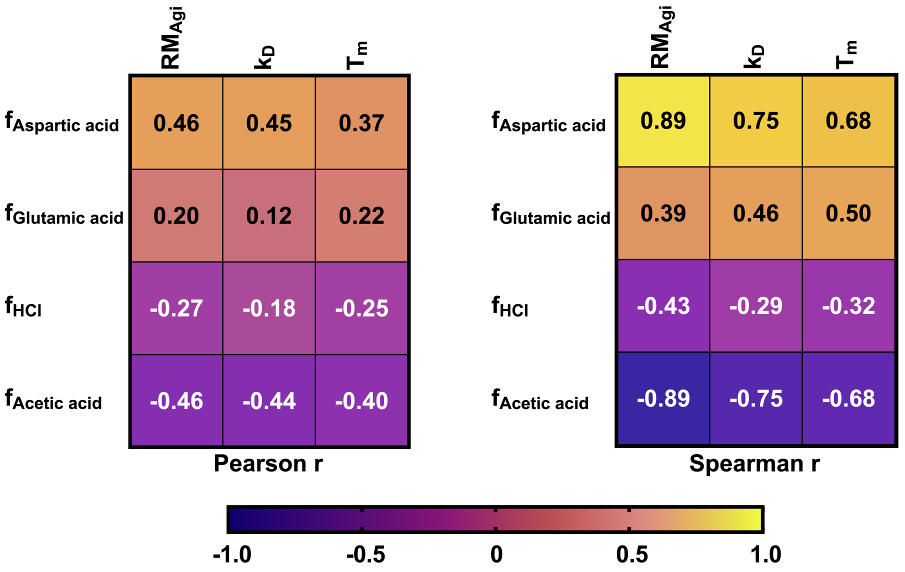

Figure S10: Pearson (left) and Spearman (right) correlation matrix of all biophysical target properties (RM_{Agi}, k_D, and T_m) and the acid fractions. Despite observable trends in acid-specific effects on biophysical properties (ranking: aspartic acid > glutamic acid > HCl > acetic acid), no strong conclusions can be drawn due to the substantial reduction in arginine concentration after initialization, which concurrently reduced acid levels.

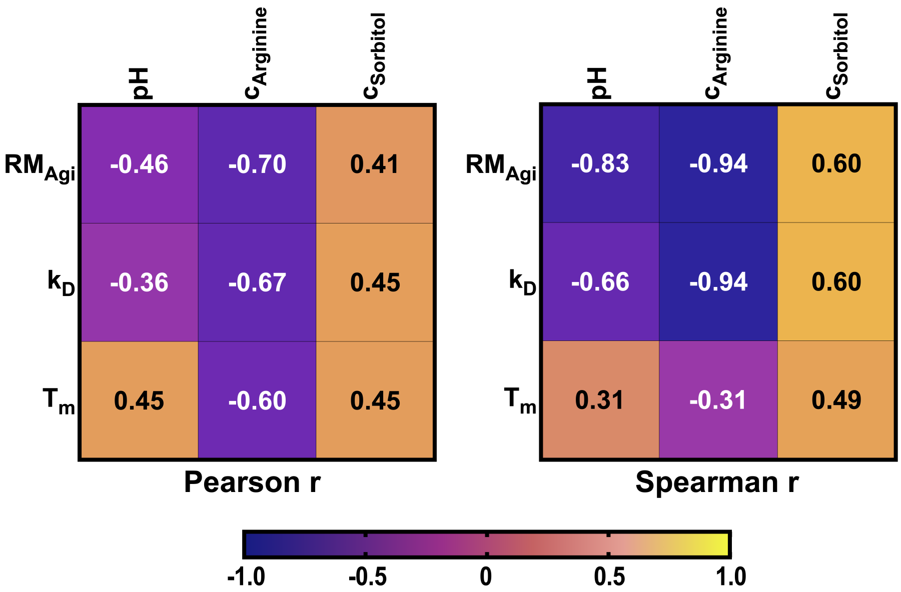

Figure S11: Pearson (left) and Spearman (right) correlation matrix of all biophysical target properties (RM_{Agi}, k_D, and T_m) and the most relevant excipients/properties (pH, $c_{Arginine}$, and $c_{Sorbitol}$). Pearson correlation measures the strength of a linear relationship between variables, while Spearman correlation assesses the strength of a monotonic relationship based on ranks and is more robust to non-normal data and outliers.

High Concentration Analysis

Viscosity and Opalescence Measurements

Samples were buffer exchanged and concentrated with Amicon centrifugal filters and the target concentration of 125 mg mL^{-1} was reached within ±5% variation. Protein concentration was determined with A280 (Dropsense 96, Unchained Labs) using a theoretical extinction coefficient of 213.38 cm^{-1} M^{-1}. Viscosity was measured with an automated viscometer (VROC initium one plus, Rheosense) at 20 ℃, a sheer rate of 2.500 s^{-1} and averaging of five repeat measurements. Opalescence was measured at 20 ℃ using a light scattering method described in Kingsbury et al. (J. Pharm. Sci. 2021, 110, 3176-3182). In addition, the vehicle opalescence and viscosity readings were determined and subtracted from the sample measurement results.

SAXS Measurements

SAXS experiments were conducted using Xeuss from Xenocs with GeniX 3D Cu High Flux Very Long Focus (HFVL) X-ray source with motorized detector with x-rays wavelength of 1.5418 Å and the distance from the sample to the detector of 600 mm. All the samples and corresponding buffers were prepared in 96-well plate and 17 µL were automatically injected with a sample handling robot. All the measurements were performed at 25 ℃. Five frames with 120 s of exposure time were collected for each sample and automatically averaged using RAW software, resulting in q-range from 0.000 25-0.451 04 Å. Protein scattering was obtained by subtracting averaged buffer scattering from the averaged sample scattering and normalized to concentration using ATSAS 3.04. The absolute scaling method was performed by comparison to a scattering of internal standard (human insulin). Radius of gyration R_g and maximum dimension D_{max} were derived from pair distance distribution function (p(r)). The scattering intensity, S(q), for each sample was calculated by dividing the SAXS scattering of each sample by the SAXS scattering of a 1 mg L^{-1} sample prepared in the same buffer. The value of S(0) was obtained by extrapolating the data to y=0 using the first 25 data points.

Opalescence, Viscosity, and SAXS Measurement Results

Table S4: Opalescence and viscosity measurement results (vehicle opalescence/viscosity subtracted). For the opalescence the 95% confidence interval is provided (n=2). The control formulation contains only histidine (10mM, pH 6).

Formulation	Opalescence [NTU]	Viscosity [mPa*s]
Control	18.0 ± 4.5	5.3
31	17.1 ± 0.8	10.6
30	20.8 ± 4.3	6.1
24	23.8 ± 0.7	7.8
25	13.9 ± 3.2	6.8
9	29.5 ± 1.7	7.1
6	15.1 ± 3.9	3.0
21	15.3 ± 1.2	6.4
4	8.2 ± 0.4	3.0

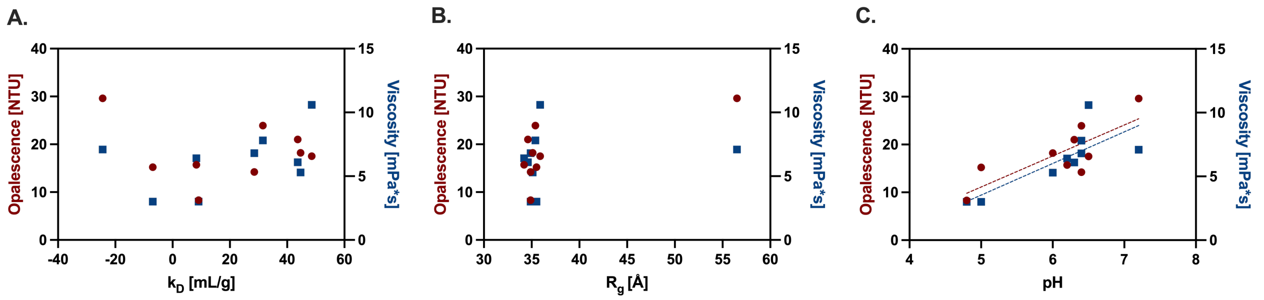

Figure S12: Opalescence and viscosity measurements of selected formulations at high mAb concentration (125 mg mL^{-1}). **A.** No correlation was observed between k_D, measured at low mAb concentrations (1-7 mg mL^{-1}), and opalescence or viscosity at high mAb concentration. **B.** The R_g measured by SAXS did not correlate with the opalescence or viscosity measurements. Increased repulsion at high mAb concentration led to an underestimation of R_g. **C.** Formulations at lower pH show reduced viscosity and opalescence compared to those at higher pH.

Table S5: Overview of the SAXS scattering data at t_0.

Formulation	Conc. [mg/mL]	R_g [Å]	D_{max} [Å]	M_w [kDa]*	Oligomeric state*	Relative S(0)**
Control	1	57.8	185	127.2	0.9	-
	10	50	148	122.5	0.8	0.85
	25	48.2	146	127.3	0.9	0.51
	50	42.7	130	93.9	0.6	0.36
	75	39.4	125	72.3	0.5	0.32
	100	35.1	116	54.4	0.4	0.3
	120	33.7	116	46.4	0.3	0.27
31	1	55	165	131.8	0.9	-
	10	50.7	150	121.8	0.8	0.61
	25	49.6	148	117.2	0.8	0.3
	50	45.2	140	90.1	0.6	0.21
	75	39.2	122	62.4	0.4	0.18
	100	35.9	118	48.5	0.3	0.16
	129.7	31.7	114	36	0.2	0.13
30	1	54.4	165.0	116.9	0.8	-
	10.0	51.1	150.0	110.6	0.8	0.64
	25.0	48.7	144.0	110.1	0.8	0.32
	50.0	44.6	137.0	89.4	0.6	0.18
	75.0	40.1	124.0	66.4	0.5	0.17
	100.0	34.6	115.0	47	0.3	0.15
	125.8	31.1	110.0	36.7	0.3	0.13
24	1	57.1	175.0	104.5	0.7	-
	10.0	49.8	147.0	94.2	0.6	0.75
	25.0	49.1	146.0	98.6	0.7	0.36
	50.0	44.4	134.0	76	0.5	0.21
	75.0	40.5	126.0	57.8	0.4	0.18
	100.0	35.4	118.0	42.7	0.3	0.15
	126.7	-	-	-	-	-
25	1	56.8	175.0	113.8	0.8	-
	10.0	52	157.0	103	0.7	0.71
	25.0	49.6	150.0	112.1	0.8	0.35
	50.0	45.5	137.0	89.7	0.6	0.22
	75.0	39.2	122.0	60.2	0.4	0.18
	100.0	34.9	115.0	46.3	0.3	0.16
	124.6	31	112.0	32.7	0.2	0.14
9	1	56.9	190.0	132	0.9	-
	10.0	54.1	165.0	131.1	0.9	0.96
	25.0	57.1	195.0	133.8	0.9	0.97
	50.0	57.9	205.0	121.8	0.8	0.88
	75.0	57.7	215.0	100.3	0.7	0.71
	100.0	56.5	230.0	79.2	0.5	0.56
	127.8	52.6	220.0	56.4	0.4	0.40
6	1	58.4	185.0	122.6	0.8	-
	10.0	50.8	152.0	102.5	0.7	1.08
	25.0	47.3	143.0	91.5	0.6	0.97
	50.0	43.4	135.0	68.4	0.5	0.70
	75.0	39.3	122.0	51.7	0.4	0.53
	100.0	35.5	116.0	39	0.3	0.40
	127.9	32.1	115.0	29.1	0.2	0.29
21	1	55.3	170.0	105.2	0.7	-
	10.0	50.4	155.0	99.8	0.7	0.56
	25.0	48.8	145.0	94.2	0.6	0.26
	50.0	43.6	132.0	71.7	0.5	0.15
	75.0	39.3	124.0	52.9	0.4	0.11
	100.0	34.2	118.0	37.3	0.3	0.10
	130.4	-	-	-	-	-
4	1	56.9	175.0	112.5	0.8	-
	10.0	49.2	145.0	97.9	0.7	0.29
	25.0	46.1	136.0	77.8	0.5	0.22
	50.0	-	-	-	-	-
	75.0	36.2	120.0	40.1	0.3	0.10
	100.0	34.9	122.0	32	0.2	0.07
	128.1	27.1	110.0	19.7	0.1	0.05

*Averaged M_w and oligomeric state are underestimated due to high repulsion. **S(q) was calculated using 1 mg mL^{-1} scattering curve, assuming I(q)=P(q).
